# Efficacy and Safety of *Cordyceps militaris* as an Adjuvant to Duloxetine in the Treatment of Insomnia in Patients With Depression: A 6-Week Double- Blind, Randomized, Placebo-Controlled Trial

**DOI:** 10.3389/fpsyt.2021.754921

**Published:** 2021-11-11

**Authors:** Jiaojiao Zhou, Xu Chen, Le Xiao, Jingjing Zhou, Lei Feng, Gang Wang

**Affiliations:** The National Clinical Research Center for Mental Disorders and Beijing Key Laboratory of Mental Disorders, Beijing Anding Hospital, The Advanced Innovation Center for Human Brain Protection, Capital Medical University, Beijing, China

**Keywords:** depression, insomnia, *Cordyceps militaris*, duloxetine, efficacy, safety

## Abstract

**Background:** Insomnia is a common clinical manifestation in patients with depression. Insomnia is not only a depression symptom but also an independent risk factor for recurrence. *Cordyceps militaris* (*C. militaris*) is thought to have the potential to treat insomnia. This study aimed to examine the efficacy and safety of duloxetine with *C. militaris* in improving sleep symptoms in patients with depression.

**Methods:** This study was a single-center, randomized, double-blind, placebo-controlled study that recruited outpatients admitted to Beijing Anding hospital from January 2018 to January 2019. Major depressive disorder (MDD) with insomnia was diagnosed according to the Diagnostic and Statistical Manual of Mental Disorders (DSM-IV-TR) criteria and Mini-International Neuropsychiatric Interview (M.I.N.I.). Eligible subjects will be randomly assigned to two treatment groups in a 1:1 ratio, and receive treatment and follow-up of about 6 weeks of duloxetine plus *Cordyceps militaris* or placebo, respectively. The severity of depression and insomnia was evaluated at baseline and at 1, 2, 4, and 6 weeks using the 17-item Hamilton Depression Scale (HAMD-17) and Athens Insomnia Scale (AIS).

**Results:** A total of 59 subjects were included in the study (31 in the placebo group and 28 in the *C. militaris* group). 11 (18.6%) participants withdrew during the study period, 5 (17.9%) in the *C. militaris* group, and 6 (19.3%) in the placebo group. Depressive and sleep symptoms in all patients reduced over time. We found that the total scores of AIS and its subscales decreased more in the placebo group compared to the *C. militaris* group (*p* < 0.05). Secondary outcome revealed that there were no significant differences between the two groups in total HAMD-17 and its sleep factor scores (*p* > 0.05) at 1, 2, 4, and 6 weeks after treatment initiation. The incidences of adverse events were not significantly different between the two groups (all *p* > 0.05).

**Conclusion:** *C. militaris* at the current dose and duration did not improve sleep symptoms in patients with depression, but it is safe with rare side effects.

## Introduction

Major depressive disorder (MDD) is a common chronic psychiatric disorder with high morbidity, disability, and recurrence (1) that is projected to be one of the leading worldwide causes of disability by 2030 (2). Previous studies have shown that the balance between glutamate and γ-aminobutyric acid (GABA) is becoming increasingly relevant in the field of depression, thus α-amino-3-hydroxy-5-methyl-4-isoxazole propionic acid (AMPA) receptor activation is recently considered as one of the most promising approaches for antidepressant therapies (3).

Accumulating evidence suggests that ~90% of patients with depression experience insomnia (4), which is considered not only a symptom of depression but also a significant predictor of depression (5). Although depressive symptoms improve after treatment (6–8). Insomnia often remains and predicts a shorter remission and increased risk of relapse (9). Clinically, depression is frequently accompanied by sleep disorders, which often seriously affect cognitive level, social function, and depression recovery.

Currently, there are two approaches to clinical treatment of sleep disorders, pharmacological and non-pharmacological. Pharmacological treatments of insomnia mainly include benzodiazepines, non-benzodiazepines, and sedating antidepressants (10). However, long-term use of these drugs has undesirable adverse effects, such as headache, forgetfulness, fatigue, dependence, changes in sleep structure, and hangover (11). In addition, antidepressant-induced insomnia can occur and delay recovery from MDD (12). Non-pharmacological therapy mainly consists of cognitive behavioral therapy (13). Its use is supported by evidence-based medicine and recommended by treatment guidelines. However, cognitive behavioral therapy resources are relatively scarce in China and it is underutilized. Other non-pharmacological therapies include diet therapy, aromatherapy, massage, homeopathy, and light therapy but clinical evidence supporting their use is lacking (14). Although clinical studies have shown that acupuncture is effective in treating insomnia (15), the studies were not rigorous and should be treated with caution (16). Biofeedback is another alternative insomnia treatment that has been used for many years (17). Recently, both drug therapy and non-drug therapy have some problems for depression with insomnia, including side effects and poor efficacy, resulting in unsatisfactory therapeutic effect. Therefore, novel and effective approaches to insomnia treatment in MDD with fewer adverse effects are needed.

Recently, sleep mechanism studies have found that the gradual accumulation of endogenous sleep-promoting factors during wakefulness form the basis of sleep (18). Further investigations have suggested that adenosine is a significant endogenous sleep-promoting substance (19–21). Systemic administration of adenosine analogs or inhibitors of its metabolism increase non-rapid eye movement (NREM) sleep in rats (22). Adenosine is a neuromodulator formed by hydrolysis of adenosine monophosphate (AMP) or S-adenosylhomocysteine (23). It promotes sleep by reacting with one of the four types of adenosine receptors, A1, A2A, A2B, and A3 (24). Melling et al. screened plant materials associated with adenosine receptor activity to identify supplements that improve sleep quality and found that *Cordyceps militaris* (*C. militaris*) contained cordycepin (3′-deoxyadenosine), an important bioactive constituent and naturally occurring adenosine analog that has been used in traditional medicine for treating insomnia for hundreds of years (25). Its long history of use alone provides good evidence for its safety. In 2009, the Ministry of Health of the People's Republic of China issued a notice to consider *C. militaris* as a new food resource. In addition, recent studies have shown that cordycepin promotes NREM sleep in rats (26). However, there were no other studies with *C. militaris* in humans yet. This study aimed to evaluate the efficacy of cordycepin in improving insomnia in patients with depression.

## Methods

### Study Design and Participants

This double-blind, randomized, placebo-controlled clinical trial was conducted from January 1, 2018 to February 1, 2019. Adult patients diagnosed with depression were randomly allocated to receive either 6 weeks of *C. militaris* or placebo. The study was approved by the institutional review board of the Beijing Anding Hospital, Capital Medical University and registered with Clinical-Trials.gov (number ChiCTR-INR-17014074).

All participants provided written informed consent. Patients who met the following criteria were eligible: (1) age 18–65 years; (2) diagnosis of MDD with insomnia according to the Diagnostic and Statistical Manual of Mental Disorders (DSM-IV-TR) criteria and Mini-International Neuropsychiatric Interview (M.I.N.I.) (27); (3) moderate or greater severity of symptoms as indicated by a score of ≥ 17 on the Hamilton Depression Scale (HAMD-17) (28); (4) Athens Insomnia Scale (AIS) score >6 (29); and (5) no antipsychotic treatment for at least 3 months.

Exclusion criteria included the following: (1) serious comorbid cardiovascular, neurological, or other unstable medical condition; (2) suicidal ideation or attempts or aggressive behavior; (3) hepatic and renal function testing and/or electrocardiogram (ECG) beyond the normal reference range; (4) history of alcoholism or drug abuse within the previous 6 months; (5) allergy to duloxetine or *C. sinensis*; and (6) pregnancy or lactation.

### Randomization and Blinding

Eligible subjects were randomly assigned to 1 of 2 groups in a 1:1 ratio: group A, duloxetine combined with *C. militaris*; group B, duloxetine combined with placebo. Random codes were produced in advance by an independent researcher not involved in the study via computerized number generation. Intervention allocation was stored in an envelope that was opened for statistical analysis after the data collection period was completed. All study personnel, including subjects, drug dispensers, outcome assessors, data collectors, and analysts, were blinded to allocation throughout the study period.

### Treatment

A fixed dosing schedule was employed throughout the study (duloxetine capsule, 60 mg/day; *C. militaris* or placebo tablet, 3 g/day). The drug dosage was selected according to the approved drug specification. *C. militaris* or matching placebo was administered using a double-blind format, while duloxetine was administered open-label. All participants received 1 duloxetine capsule daily after breakfast. *C. militaris* or placebo was administered as three tablets daily after dinner. The concomitant use of antipsychotics, other antidepressants, Chinese medicine affecting the central nervous system, or melatonin was prohibited from the washout period to the end of treatment. If necessary, medication for insomnia was only permitted after 2 weeks from the beginning of the trial and was only allowed for use <1 week.

### Efficacy Endpoints

Treatment outcome was evaluated at baseline and weeks 1, 2, 4, and 6. The AIS is a validated 8-item self-report questionnaire that assesses insomnia symptom. It has been shown to have appropriate diagnostic utility, including a set of items for assessing nocturnal sleep disturbance and daytime dysfunction. It is a useful instrument to screening insomnia, and the diagnostic accuracy of this scale (sensitivity and specificity) was high. A sum score is calculated (range: 0–24), with lower scores indicating fewer insomnia symptoms (29). The primary outcome was mean change in total AIS score and its subscales over the treatment period with day 1 as baseline. The HAMD-17 was used to assess the severity of depressive symptoms in the past week. The Chinese version of the HAMD-17 has been validated in Chinese populations and has good psychometric characteristics. The scale contains 17 variables and each item ranged from 0 to 2 or 0 to 4. A sum score is calculated (range: 0–52), with higher scores indicating more severe depressive symptoms (28). Secondary outcome was change in total HAMD-17 score and its sleep factor score.

### Safety

Safety evaluation included physical examination and assessment of vital signs and adverse events at each follow-up visit. Adverse events were recorded, including date and time of onset, duration, severity, relationship to intervention, and action taken. Clinical laboratory tests and 12-lead ECGs were performed at screening and at the endpoint.

### Statistical Analyses

A total of 59 subjects were included in the study. Under the repeated measurement design, 80% power with a type I error of 0.05, the lowest effect size difference between the two groups was found (effect size = 0.3).

Efficacy analyses were performed on the intention-to-treat population who completed baseline and at least one evaluation after treatment. Linear mixed-effect models were used to compare differences in treatment outcome between the two groups. The model was established using time and group as categorical fixed factors and random intercepts. Baseline scores were included as covariates. An autoregressive covariance structure that produced the smallest Bayesian Information Criterion was applied. The safety analysis population included all randomized participants who took at least one dose of study medication. Categorical variables were analyzed using the Chi-square test. The Tukey test was used to detect differences in continuous variables between the two groups. Two-tailed *p* < 0.05 was considered significant. All analyses were conducted using SPSS software version 22.0.

## Results

### Participant Characteristics

**Figure 1:**
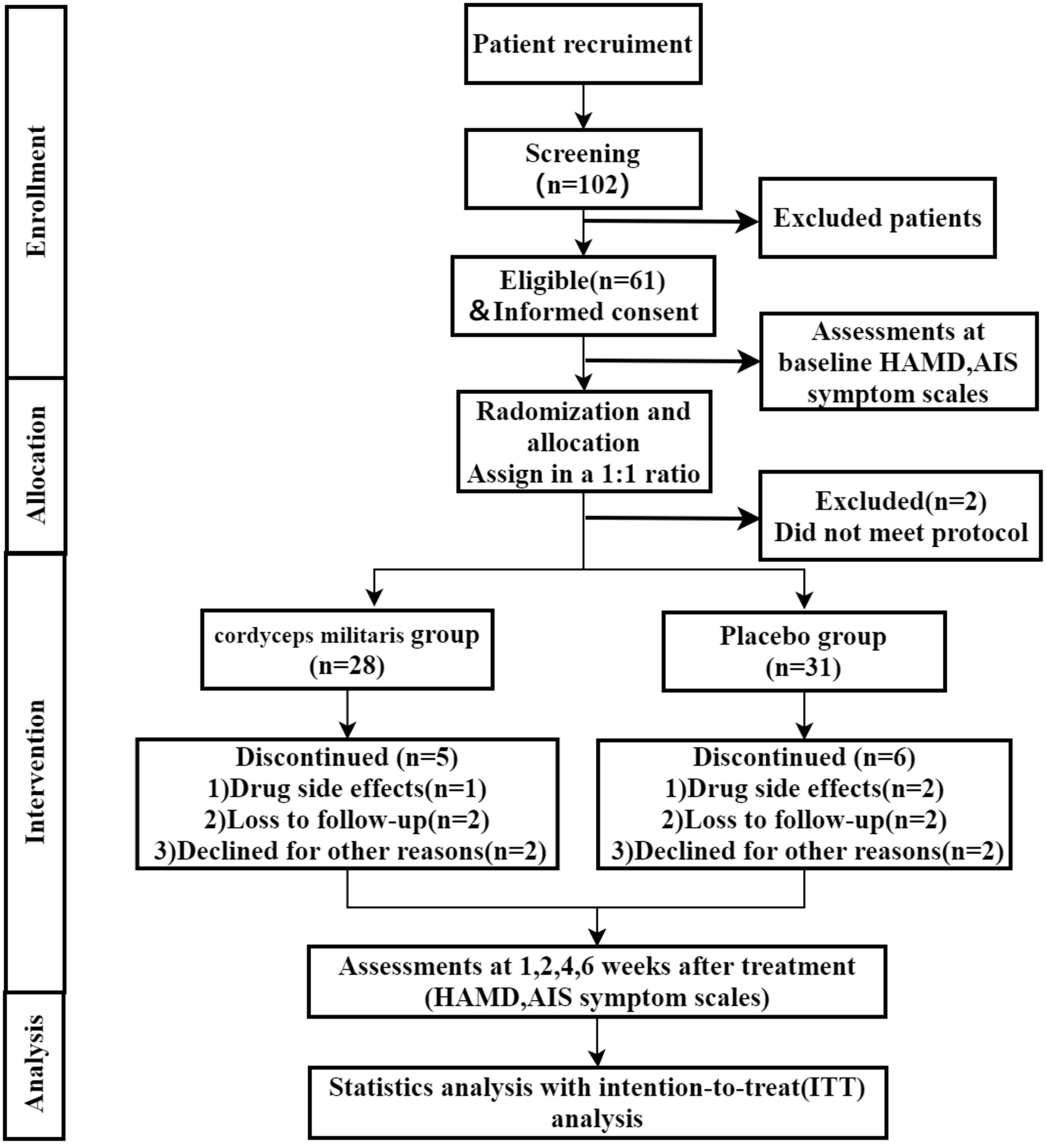
Study flowchart.

A total of 61 participants were recruited and randomized, among which 2 subject who did not meet the inclusion criteria were excluded. So, there were 59 patients included in this study (31 in the placebo group and 28 in the *C. militaris* group). The study flowchart is shown in Figure 1. Patient characteristics were well-balanced between the two groups (Table 1). 11 (18.6%) participants withdrew during the study period, 5 (17.9%) in the *C. militaris* group, and 6 (19.3%) in the placebo group. Withdrawal reasons are provided in Figure 1.

**Table 1:** Sociodemographic data and clinical characteristics of the participants.

**Variables**	**Placebo group**	***Cordyceps militaris* group**	***p*-value**
Age, year	30.87 ± 8.76	30.21 ± 7.52	0.760
Gender, n (%)			0.167
Male	8 (25.8%)	12 (42.9%)	
Female	23 (74.2%)	16 (57.1%)	
Body high, cm	166.4 ± 7.8	166.5 ± 7.10	0.922
Body weight, kg	59.2 ± 11.6	63.3 ± 10.6	0.158
Educational level, n (%)			0.812
Beneath college degree	12 (38.7%)	10 (35.7%)	
College degree or higher	19 (61.3%)	18 (64.3%)	
Employment status, n (%)			0.915
Yes	24 (77.4%)	22 (78.6%)	
No	07 (22.6%)	06 (21.4%)	
Marital status, n (%)			0.985
Married	11 (35.5%)	10 (47.6%)	
Single	20 (64.5%)	18 (64.3%)	
First-episode subjects, n (%)			0.648
Yes	17 (42.4%)	17 (60.7%)	
No	14 (45.2%)	11 (39.3%)	
Psychiatric family history, n (%)
Yes	3 (9.7%)	3 (10.7%)	
No	28 (90.3%)	25 ()	
Baseline total HAMD^a^ score	23.77 ± 4.28	24.89 ± 4.01	0.306
Baseline total AIS^b^ score	13.61 ± 2.90	14.71 ± 3.26	0.175

a *HAMD, Hamilton Depression Scale*.

b *AIS, Athens Insomnia Scale*.

### Primary Outcomes

**Figure 2:**
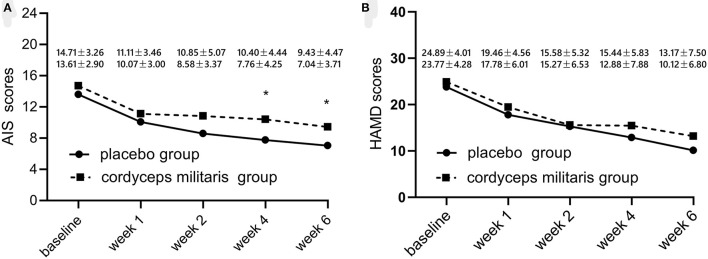
Changes in sleep quality **(A)** and HAMD score **(B)** with treatment from baseline to 6 weeks after treatment initiation. The placebo group is represented by the dotted line and the *C. militaris* group is represented by the solid line. *p < 0.05.

Changes in overall AIS score and subscale scores are illustrated in Table 2 and Figure 2A. The linear mixed-effect model showed significant slope differences between the two groups for total AIS score (*F* = 6.22, *p* < 0.05), awakenings during the night (*F* = 16.02, *p* < 0.001), final awakening (*F* = 9.42, *p* < 0.05), total sleep duration (*F* = 6.87, *p* < 0.01), sleep quality (*F* = 9.70, *p* < 0.05), and functioning capacity during the day (*F* = 10.06, *p* < 0.01). Between-group comparisons showed a significantly greater reduction in AIS score in the placebo group compared to the *C. militaris* group (*p* < 0.05). The AIS score markedly decreased over the course of treatment in each group (*p* < 0.01).

**Table 2:** Changes of AIS factors and total scores at baseline, week 1, week 2, week 4, and week 6.

**Variables**	**Placebo group**	***Cordyceps militaris* group**	**Overall analysis^a^**	
			***F***	***p*-value**
Total AIS scores^b^			6.22	0.015^*^
Baseline	13.6 ± 12.89	14.71 ± 3.26		
Week 1	10.07 ± 3.00	11.11 ± 3.46		
Week 2	8.58 ± 3.37	10.85 ± 5.07		
Week 4	7.76 ± 4.25^*^	10.40 ± 4.44^*^		
Week 6	7.04 ± 3.71^*^	9.43 ± 4.47^*^		
Sleep induction			0.41	0.521
Baseline	1.87 ± 0.81	1.86 ± 0.93		
Week 1	1.19 ± 0.62	1.18 ± 0.72		
Week 2	1.31 ± 0.74	1.27 ± 0.96		
Week 4	1.28 ± 0.79	1.40 ± 0.96		
Week 6	1.04 ± 0.68	1.13 ± 0.87		
Awakenings during the night			16.02	<0.001^*^
Baseline	1.52 ± 0.62	1.68 ± 0.82		
Week 1	1.26 ± 0.71	1.46 ± 0.69		
Week 2	0.96 ± 0.72^*^	1.46 ± 0.71^*^		
Week 4	0.92 ± 0.70^*^	1.40 ± 0.82^*^		
Week 6	0.68 ± 0.56^*^	1.30 ± 0.82^*^		
Final awakening			9.42	0.002^*^
Baseline	1.16 ± 0.81	1.89 ± 0.63		
Week 1	1.11 ± 0.70^*^	1.57 ± 0.69^*^		
Week 2	1.04 ± 0.66	1.32 ± 0.85		
Week 4	0.76 ± 0.66^*^	1.32 ± 0.85^*^		
Week 6	0.76 ± 0.60	1.13 ± 0.87		
Total sleep duration			6.87	0.009^*^
Baseline	1.61 ± 0.62	2.93 ± 0.90		
Week 1	0.87 ± 0.85	1.07 ± 0.90		
Week 2	0.74 ± 0.73	1.11 ± 0.92		
Week 4	0.61 ± 0.80^*^	1.21 ± 0.92^*^		
Week 6	0.71 ± 0.78	1.04 ± 0.92		
Sleep quality			9.70	0.020^*^
Baseline	2.06 ± 0.51	2.21 ± 0.57		
Week 1	1.10 ± 0.70	1.32 ± 0.67		
Week 2	0.94 ± 0.73^*^	1.39 ± 0.88^*^		
Week 4	0.94 ± 0.72	1.36 ± 0.91		
Week 6	0.68 ± 0.70	1.07 ± 0.90		
Well-being during the day			0.76	0.386
Baseline	1.84 ± 0.82	1.96 ± 0.64		
Week 1	1.00 ± 0.68	1.32 ± 0.82		
Week 2	0.81 ± 0.70	1.04 ± 0.79		
Week 4	0.68 ± 0.75	0.86 ± 0.85		
Week 6	0.58 ± 0.56	0.61 ± 0.73		
Functioning capacity during the day			10.06	0.002^*^
Baseline	1.84 ± 0.78	1.89 ± 0.74		
Week 1	1.13 ± 0.85^*^	1.54 ± 0.64^*^		
Week 2	0.97 ± 0.71^*^	1.39 ± 0.83^*^		
Week 4	0.74 ± 0.77	1.11 ± 0.74		
Week 6	0.71 ± 0.69	1.00 ± 0.77		
Sleepiness during the day			1.30	0.256
Baseline	1.29 ± 0.86	1.29 ± 0.66		
Week 1	1.58 ± 0.96	1.64 ± 0.87		
Week 2	0.97 ± 0.84	1.43 ± 0.96		
Week 4	0.90 ± 0.87	1.07 ± 0.86		
Week 6	1.0 ± 0.97	1.11 ± 0.99		

a *Overall statistical significance was analyzed using a linear mixed-effect model analysis. Between-group differences were further evaluated using one-way analysis of variance (ANOVA)*.

b *AIS, Athens Insomnia Scale*.

* *Statistically significant*.

### Secondary Outcomes

Table 3 shows the differences in HAMD and sleep factor scores between the two groups. The linear mixed-effect model showed that HAMD score did not significantly differ between the two groups from baseline to the end of treatment. The subcomponent scores also showed no significant differences between the two groups (*F* = 1.65, *p* > 0.05). Figure 2B shows that the HAMD score was not significantly different between the two groups at different time points. However, the HAMD score improved over time for each group (*p* < 0.01).

**Table 3:** Changes of HAMD sleep factors and total scores at baseline, week 1, week 2, week 4, and week 6.

**Variables**	**Placebo group**	***Cordyceps militaris* group**	**Overall analysis^a^**	
			***F***	***p*-value**
Total HAMD-17 scores^b^			1.65	0.204
Baseline	23.77 ± 4.28	24.89 ± 4.01		
Week 1	17.78 ± 6.01	19.46 ± 4.56		
Week 2	15.27 ± 6.53	15.58 ± 5.32		
Week 4	12.88 ± 7.88	15.44 ± 5.83		
Week 6	10.12 ± 6.80	13.17 ± 7.50		
Sleep disorders			0.28	0.600
Baseline	4.81 ± 0.70	5.04 ± 0.64		
Week 1	3.04 ± 1.60	3.18 ± 1.66		
Week 2	3.19 ± 1.60	2.96 ± 1.73		
Week 4	2.68 ± 1.39	3.16 ± 1.89		
Week 6	2.48 ± 1.39	3.56 ± 1.93		

a *Overall statistical significance was analyzed using a linear mixed-effect model analysis. Between-group differences were further evaluated using one-way analysis of variance (ANOVA)*.

b *HAMD-17,17 items of the Hamilton Depression Scale*.

### Adverse Events

The incidence of adverse events is shown in Supplementary Table 1. No severe adverse events were reported. The incidence of nausea, drowsiness, palpitation, dizziness, loss of appetite, vomiting, and excessive sweating was not significantly different between the two groups.

## Discussion

To the best of our knowledge, this preliminary study is the first double-blind, randomized, placebo-controlled clinical trial investigating the efficacy, tolerability, and safety of *C. militaris* as an additional treatment for insomnia symptoms in patients with MDD. *C. militaris* is an edible medicine product with confirmed anticancer, antibacterial, antiviral, immunomodulatory, neuroprotective, and antioxidant effects based on previous research (30, 31). Its main ingredient is an adenosine analog that has the potential to improve sleep through its physiological action on adenosine receptors; however, the mechanism remains controversial. *C. militaris* contained cordycepin (3′-deoxyadenosine). A previous study have proved that Cordycepin could produce a rapid and robust antidepressant Effect via enhancing pre-frontal AMPA receptor signaling pathway in male CD-1 mice (32). Although previous basic science studies of *C. militaris* have shown that it can extend the period of NREM sleep via association with adenosine receptors (26), no clinical trials have been conducted until now. Besides, there were no other studies with *C. militaris* in humans yet.

This study found that insomnia symptoms as measured by total AIS score were not improved by the addition of *C. militaris* to a 6-week course of duloxetine compared to placebo. However, the efficacy of duloxetine plus placebo was better than duloxetine plus *C. militaris* as measured by changes in total AIS and some subscale scores (awakenings during the night, final awakening, total sleep duration, sleep quality, and functioning capacity during the day). This interesting result may be due to the fact that this is a single-center exploratory study with limited sample size. Multi-center studies with large sample sizes in future might have different and more credible results.

In contrast to patient-reported insomnia symptoms, there was no significant difference in physician-assessed sleep improvement (HAMD-17 sleep factor score). This contrast suggests that reliance on subjective patient symptom assessment may be insufficient when evaluating insomnia and that objective sleep monitoring, such as sleep electroencephalography (EEG), is needed. In addition, the proportion of patients with first-episode depression in this study was relatively high. A previous meta-analysis found that the effectiveness of antidepressant single-drug treatment for first-episode depression is as high as 64.5% (31), and for most patients with first-episode depression, sleep difficulties improve along with reduction of depression. In this study, depressive symptoms significantly decreased in both groups after the first week of treatment, which we attributed to duloxetine. Furthermore, cordycepin has a short half-life in humans, unlike animals, and the dose and frequency of drug administration may also affect the results (33).

In addition, this study demonstrated no significant difference between the effects of 6 weeks of treatment with duloxetine plus *C. militaris* or duloxetine plus placebo on the secondary endpoints (i.e., change in total HAMD-17 and its sleep factor scores) or in the MDD treatment response. A significant improvement in HAMD-17 scores was found in both groups, consistent with previous findings (34).

The safety of *C. militaris* treatment for insomnia in patients with MDD is worth discussion. Minimal adverse events were reported for both *C. militaris* and placebo and treatment with duloxetine plus *C. militaris* did not increase the study withdrawal rate compared with duloxetine plus placebo. In addition, since 2009, when the ministry of health of China approved *C. militaris* as a new resource food (ministry of health announcement no. 3, 2009), it has been widely used and no adverse reactions have been reported, consistent with the results of this study (35).

The current study has several limitations, including small sample size, high number of participants with first-episode depression and mild sleep problems, reliance on patient reporting for measuring primary outcome, and a lack of objective sleep assessment.

Future multi-center studies should employ larger samples that include a wider representation of patients with MDD with sleep problems and utilize polysomnography to monitor sleep improvement.

## Conclusion

*C. militaris* treatment at the current dose and duration is not recommended for the reduction of sleep symptoms in patients with MDD. Although *C. militaris* is safe and side effects are rare, its efficacy needs to be verified by future large-scale studies.

## Data Availability Statement

The raw data supporting the conclusions of this article will be made available by the authors, without undue reservation.

## Ethics Statement

The studies involving human participants were reviewed and approved by the Institutional Review Board of the Beijing Anding Hospital, Capital Medical University. The patients/participants provided their written informed consent to participate in this study.

## Author Contributions

GW designed the study. JJZ and XC wrote the protocol and analyzed the data. LX and JJZ commented on the protocol. JJZ and XC wrote the first draft of manuscript with input from LF. All authors commented on and approved the final manuscript.

## Funding

The study was supported by the National Key R&D Program of China (2017YFC1311100), Beijing Brain Science Research Project (Z171100000117004), and the Beijing Municipal Science and Tech Commission (D171100007017001).

## Conflict of Interest

The authors declare that the research was conducted in the absence of any commercial or financial relationships that could be construed as a potential conflict of interest.

## Publisher's Note

All claims expressed in this article are solely those of the authors and do not necessarily represent those of their affiliated organizations, or those of the publisher, the editors and the reviewers. Any product that may be evaluated in this article, or claim that may be made by its manufacturer, is not guaranteed or endorsed by the publisher.
