# Supplementary material for: Efficacy and Safety of Cordyceps militaris as an Adjuvant to Duloxetine in the Treatment of Insomnia in Patients With Depression: A 6-Week Double- Blind, Randomized, Placebo-Controlled Trial
Source: Front Psychiatry. 2021 Nov 11;12:754921. doi: 10.3389/fpsyt.2021.754921 (PMC8632006; doi:10.3389/fpsyt.2021.754921)
Supplement: Supplementary file 1 [file Table_1.DOCX]

Supplementary table 1 The incidence of adverse events in patients with depression

| Adverse event | placed group n（%） | cordyceps militaris group n（%） | p value |
| --- | --- | --- | --- |
| Any | 17（54.8） | 19（67.9%） | 0.306 |
| Nausea | 7（22.6） | 2（7.10） | 0.100 |
| [Drowsiness](javascript:;) | 5（16.1） | 6（21.4） | 0.602 |
| Palpitation | 3（9.70） | 2（7.10） | 0.727 |
| Dizziness | 3（9.70） | 4（14.3） | 0.585 |
| Loss of appetite | 2（6.50） | 1（3.60） | 0.615 |
| vomiting | 2（6.50） | 0（0.00） | 0.171 |
| [Excessive sweating](javascript:;) | 1（3.20） | 2（7.10） | 0.494 |
